# 2-[(*E*)-2-(4-Chloro­phen­yl)ethen­yl]-1-methyl­pyridinium 4-chloro­benzene­sulfonate

**DOI:** 10.1107/S1600536809021667

**Published:** 2009-06-13

**Authors:** Hoong-Kun Fun, Kullapa Chanawanno, Suchada Chantrapromma

**Affiliations:** a X-ray Crystallography Unit, School of Physics, Universiti Sains Malaysia, 11800 USM, Penang, Malaysia; b Crystal Materials Research Unit, Department of Chemistry, Faculty of Science, Prince of Songkla University, Hat-Yai, Songkhla 90112, Thailand

## Abstract

In the title salt, C_14_H_13_ClN^+^·C_6_H_4_ClO_3_S^−^, the cation exists in an *E* configuration with respect to the ethynyl bond and is approximately planar, with a dihedral angle of 3.4 (2)° between the pyridinium and benzene rings. The anion is approximately perpendicular to the cation plane, the benzene ring of the anion making dihedral angles of 89.4 (2) and 89.9 (2)°, respectively, with the pyridinium and benzene rings of the cation. In the crystal structure, the cations are linked into a chain along the *c* axis by C—H⋯Cl inter­actions. The anions are linked to the adjacent cation chains by C—H⋯O and C—H⋯Cl inter­actions, forming a two-dimensional network parallel to the *bc* plane. The crystal structure is further stabilized by C—H⋯π inter­actions. A π–π inter­action is also observed between the pyridinium ring and the benzene ring of the cation with a centroid–centroid distance of 3.668 (3) Å.

## Related literature

For bond-length data, see: Allen *et al.* (1987 ▶). For background to non-linear optical materials research, see: Koshima & Matsuura (1998 ▶); Prasad & Williams (1991 ▶); Wenseleers *et al.* (1998 ▶). For related structures, see: Chanawanno *et al.* (2008 ▶); Chantrapromma *et al.* (2007 ▶, 2008 ▶); Chantrapromma, Chanawanno & Fun (2009 ▶); Chantrapromma, Jansrisewangwong *et al.* (2009 ▶). For the stability of the temperature controller used in the data collection, see: Cosier & Glazer (1986 ▶).
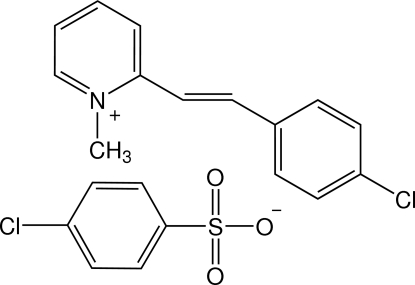


## Experimental

### 

#### Crystal data

C_14_H_13_ClN^+^·C_6_H_4_ClO_3_S^−^
*M* *_r_* = 422.32
Monoclinic, 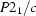

*a* = 7.9018 (7) Å
*b* = 18.5102 (17) Å
*c* = 12.6818 (12) Å
β = 93.942 (7)°
*V* = 1850.5 (3) Å^3^
*Z* = 4
Mo *K*α radiation
μ = 0.49 mm^−1^
*T* = 100 K
0.19 × 0.17 × 0.09 mm

#### Data collection

Bruker APEXII CCD area-detector diffractometer
Absorption correction: multi-scan (**SADABS**; Bruker, 2005 ▶) *T*_min_ = 0.913, *T*_max_ = 0.959
19968 measured reflections
4249 independent reflections
2426 reflections with *I* > 2σ(*I*)
*R*_int_ = 0.123

#### Refinement

*R*[*F* ^2^ > 2σ(*F* ^2^)] = 0.085
*wR*(*F* ^2^) = 0.215
*S* = 1.05
4249 reflections
245 parameters
H-atom parameters constrained
Δρ_max_ = 0.82 e Å^−3^
Δρ_min_ = −0.58 e Å^−3^

### 

Data collection: *APEX2* (Bruker, 2005 ▶); cell refinement: *SAINT* (Bruker, 2005 ▶); data reduction: *SAINT*; program(s) used to solve structure: *SHELXTL* (Sheldrick, 2008 ▶); program(s) used to refine structure: *SHELXTL*; molecular graphics: *SHELXTL*; software used to prepare material for publication: *SHELXTL* nd *PLATON* (Spek, 2009 ▶).

## Supplementary Material

Crystal structure: contains datablocks global, I. DOI: 10.1107/S1600536809021667/is2430sup1.cif

Structure factors: contains datablocks I. DOI: 10.1107/S1600536809021667/is2430Isup2.hkl

Additional supplementary materials:  crystallographic information; 3D view; checkCIF report

## Figures and Tables

**Table 1:** Hydrogen-bond geometry (Å, °)

*D*—H⋯*A*	*D*—H	H⋯*A*	*D*⋯*A*	*D*—H⋯*A*
C1—H1*A*⋯Cl2^i^	0.93	2.81	3.572 (5)	140
C3—H3*A*⋯O1^ii^	0.93	2.51	3.411 (6)	163
C6—H6*A*⋯O3	0.93	2.55	3.467 (6)	168
C7—H7*A*⋯O2^iii^	0.93	2.48	3.395 (6)	166
C13—H13*A*⋯O3	0.93	2.48	3.413 (6)	178
C14—H14*A*⋯O2^iv^	0.96	2.44	3.292 (6)	148
C14—H14*C*⋯O3	0.96	2.48	2.995 (6)	114
C16—H16*A*⋯O2	0.93	2.58	2.936 (6)	103
C19—H19*A*⋯O2^v^	0.93	2.29	3.216 (6)	178
C10—H10*A*⋯*Cg*3^vi^	0.93	2.71	3.632 (6)	172
C12—H12*A*⋯*Cg*3	0.93	2.75	3.614 (6)	154

Symmetry codes: (i) 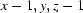; (ii) 
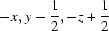
; (iii) 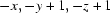; (iv) 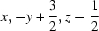; (v) 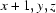; (vi) 
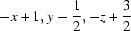
. *Cg*3 is the centroid of the C15–C20 ring.

**Fig. 1.**
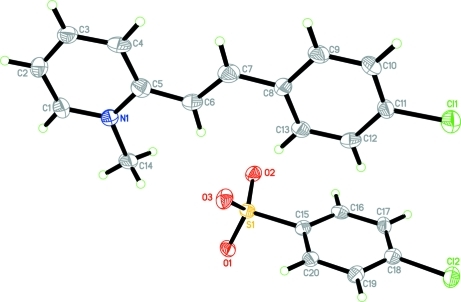
The molecular structure of the title compound, with 50% probability displacement ellipsoids and the atom-numbering scheme.

**Fig. 2.**
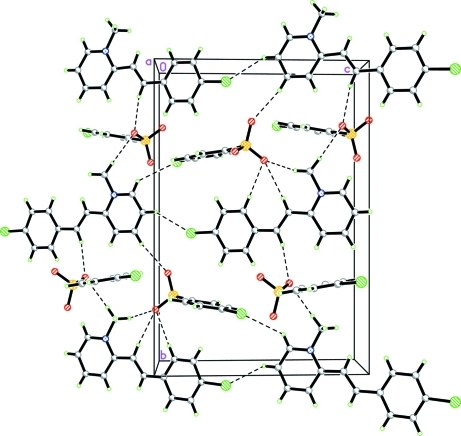
The crystal packing of the title compound viewed down the a axis. Weak C—H···O and C—H···Cl interactions are shown as dashed lines.

## supplementary crystallographic information

### Comment

To search for new materials capable for nonlinear optical (NLO) applications, many studies have focused on organic molecules containing highly polarizable π-conjugated backbones (Wenseleers *et al.*, 1998). For second order NLO compounds, an electron donor and an acceptor group are attached to both ends of this backbone to create an asymmetric "push-pull" system (Prasad & Williams, 1991). In addition, due to the inherently second-order NLO character of noncentrosymmetric organic compounds, the x-ray structure determination is a very important method to determine their NLO properties (Koshima & Matsuura, 1998). During the course of our exploring for new organic NLO materials, we have previously synthesized and reported a number of the crystal structures of pyridinium derivatives (Chanawanno *et al.*, 2008; Chantrapromma *et al.*, 2007, 2008; Chantrapromma, Chanawanno & Fun, 2009; Chantrapromma, Jansrisewangwong *et al.*, 2009). The title compound (I) has been synthesized and its crystal structure was undertaken in order to establish the conformation of the various groups and its crystal packing. The title compound crystallized in centrosymmetric space group *P*2_1_/*c* which precluded the second-order nonlinear optical properties.

In the molecule of the title compound, C_14_H_13_ClN^+^.C_6_H_4_ClO_3_S^-^ (Fig. 1), the cation exists in an *E* configuration with respect to the C6═C7 double bond [1.339 (7) Å] and the torsion angle of C5—C6—C7—C8 = 178.2 (4)°. The cation is almost planar with the dihedral angle between the pyridinium and benzene rings of the cation being 3.4 (2)°. The orientation of the anion is perpendicular with respect to the cation which is reflected by the dihedral angles between the benzene ring of the anion and the pyridinium and benzene rings of the cation being 89.4 (2)° and 89.9 (2)°. The Cl^-^ ions are coplanar with the attached benzene rings. The bond distances in both cation and anion have normal values (Allen *et al.*, 1987) and comparable with the closely related compounds (Chanawanno *et al.*, 2008; Chantrapromma *et al.*, 2007, 2008; Chantrapromma, Chanawanno & Fun, 2009; Chantrapromma, Jansrisewangwong *et al.*, 2009).

In the crystal packing (Fig. 2), all O atoms of the sulfonate group are involved in weak C—H···O interactions (Table 1). The cations and anions are individually arranged alternatively with the cations being linked into chains along the *c* axis by C—H···Cl weak interaction (Table 1). The anions are linked to the adjacent cations chains by C—H···O and C—H···Cl weak interactions forming a 2D network parallel to the *bc* plane. The crystal structure is further stabilized by C—H···π interactions (Table 1). A π–π interaction is also observed with the Cg_1_···Cg_2_ distance of 3.668 (3) Å (symmetry code: -x, 1-y, 1-z); Cg_1_, Cg_2_ and Cg_3_ are the centroids of C1–C5/N1, C8–C13 and C15–C20, respectively.

### Experimental

2-[(*E*)-2-(4-Chlorophenyl)ethenyl]-1-methylpyridinium iodide (0.24 g, 0.67 mmol) which was prepared according to the previous report (Chanawanno *et al.*, 2008) was mixed (1:1 molar ratio) with silver(I) 4-chlorobenzenesulfonate (0.20 g, 0.67 mmol) (Chantrapromma *et al.*, 2007) in methanol solution (100 ml). The mixture solution was stirred for 30 min, the precipitate of silver iodide which formed was filtered and the filtrate was evaporated to give the title compound as an orange solid. Orange needle-shaped single crystals of the title compound suitable for *x*-ray structure determination were recrystallized from methanol by slow evaporation at room temperature over a few weeks (m.p. 485-487 K).

### Refinement

All H atoms were positioned geometrically and allowed to ride on their parent atoms, with d(C-H) = 0.93 Å for aromatic and CH and 0.96 Å for CH_3_ atoms. The *U*_iso_ values were constrained to be 1.5*U*_eq_ of the carrier atom for methyl H atoms and 1.2*U*_eq_ for the remaining H atoms. A rotating group model was used for the methyl groups. The highest residual electron density peak is located at 0.91 Å from Cl1 and the deepest hole is located at 0.91 Å from S1.

### Crystal data

**Table:**

C_14_H_13_ClN^+^·C_6_H_4_ClO_3_S^−^	*F*(000) = 872
*M**_r_* = 422.32	*D*_x_ = 1.516 Mg m^−^^3^
Monoclinic, *P*2_1_/*c*	Melting point = 485–487 K
Hall symbol: -P 2ybc	Mo *K*α radiation, λ = 0.71073 Å
*a* = 7.9018 (7) Å	Cell parameters from 4249 reflections
*b* = 18.5102 (17) Å	θ = 2.0–27.5°
*c* = 12.6818 (12) Å	µ = 0.49 mm^−^^1^
β = 93.942 (7)°	*T* = 100 K
*V* = 1850.5 (3) Å^3^	Needle, orange
*Z* = 4	0.19 × 0.17 × 0.09 mm

### Data collection

**Table:**

Bruker APEXII CCD area-detector diffractometer	4249 independent reflections
Radiation source: sealed tube	2426 reflections with *I* > 2σ(*I*)
graphite	*R*_int_ = 0.123
φ and ω scans	θ_max_ = 27.5°, θ_min_ = 2.0°
Absorption correction: multi-scan (*SADABS*; Bruker, 2005)	*h* = −10→10
*T*_min_ = 0.913, *T*_max_ = 0.959	*k* = −24→23
19968 measured reflections	*l* = −15→16

### Refinement

**Table:**

Refinement on *F*^2^	Primary atom site location: structure-invariant direct methods
Least-squares matrix: full	Secondary atom site location: difference Fourier map
*R*[*F*^2^ > 2σ(*F*^2^)] = 0.085	Hydrogen site location: inferred from neighbouring sites
*wR*(*F*^2^) = 0.215	H-atom parameters constrained
*S* = 1.05	*w* = 1/[σ^2^(*F*_o_^2^) + (0.0922*P*)^2^ + 0.5785*P*] where *P* = (*F*_o_^2^ + 2*F*_c_^2^)/3
4249 reflections	(Δ/σ)_max_ = 0.001
245 parameters	Δρ_max_ = 0.82 e Å^−^^3^
0 restraints	Δρ_min_ = −0.58 e Å^−^^3^

### Special details

**Table:**

Experimental. The crystal was placed in the cold stream of an Oxford Cryosystems Cobra open-flow nitrogen cryostat (Cosier & Glazer, 1986) operating at 100.0 (1) K.
Geometry. All esds (except the esd in the dihedral angle between two l.s. planes) are estimated using the full covariance matrix. The cell esds are taken into account individually in the estimation of esds in distances, angles and torsion angles; correlations between esds in cell parameters are only used when they are defined by crystal symmetry. An approximate (isotropic) treatment of cell esds is used for estimating esds involving l.s. planes.
Refinement. Refinement of F^2^ against ALL reflections. The weighted R-factor wR and goodness of fit S are based on F^2^, conventional R-factors R are based on F, with F set to zero for negative F^2^. The threshold expression of F^2^ > 2sigma(F^2^) is used only for calculating R-factors(gt) etc. and is not relevant to the choice of reflections for refinement. R-factors based on F^2^ are statistically about twice as large as those based on F, and R- factors based on ALL data will be even larger.

### Fractional atomic coordinates and isotropic or equivalent isotropic displacement parameters (Å^2^)

**Table:**

	*x*	*y*	*z*	*U*_iso_*/*U*_eq_	
S1	0.14744 (15)	0.73096 (6)	0.57427 (10)	0.0223 (3)	
Cl1	0.60079 (17)	0.44645 (8)	0.83430 (11)	0.0374 (4)	
Cl2	0.76914 (16)	0.68816 (7)	0.90585 (10)	0.0315 (4)	
N1	−0.0338 (5)	0.5768 (2)	0.2267 (3)	0.0242 (9)	
O1	0.1460 (4)	0.80779 (17)	0.5473 (3)	0.0273 (8)	
O2	−0.0022 (4)	0.70865 (18)	0.6258 (3)	0.0273 (8)	
O3	0.1871 (4)	0.68462 (18)	0.4870 (3)	0.0300 (9)	
C1	−0.1248 (6)	0.5785 (3)	0.1326 (4)	0.0295 (12)	
H1A	−0.1595	0.6228	0.1040	0.035*	
C2	−0.1670 (6)	0.5162 (3)	0.0788 (4)	0.0306 (12)	
H2A	−0.2302	0.5180	0.0142	0.037*	
C3	−0.1146 (6)	0.4514 (3)	0.1215 (4)	0.0306 (13)	
H3A	−0.1406	0.4087	0.0852	0.037*	
C4	−0.0239 (6)	0.4492 (3)	0.2176 (4)	0.0312 (13)	
H4A	0.0093	0.4048	0.2464	0.037*	
C5	0.0196 (6)	0.5129 (3)	0.2734 (4)	0.0283 (12)	
C6	0.1196 (6)	0.5163 (3)	0.3718 (4)	0.0296 (12)	
H6A	0.1531	0.5615	0.3979	0.035*	
C7	0.1672 (6)	0.4577 (3)	0.4281 (4)	0.0309 (13)	
H7A	0.1299	0.4131	0.4018	0.037*	
C8	0.2740 (6)	0.4578 (3)	0.5282 (4)	0.0256 (12)	
C9	0.3296 (6)	0.3914 (3)	0.5712 (4)	0.0292 (12)	
H9A	0.2971	0.3487	0.5368	0.035*	
C10	0.4323 (6)	0.3886 (3)	0.6639 (4)	0.0314 (13)	
H10A	0.4709	0.3444	0.6909	0.038*	
C11	0.4766 (6)	0.4515 (3)	0.7157 (4)	0.0246 (11)	
C12	0.4242 (6)	0.5184 (3)	0.6760 (4)	0.0271 (12)	
H12A	0.4562	0.5607	0.7116	0.032*	
C13	0.3237 (6)	0.5208 (3)	0.5825 (4)	0.0268 (12)	
H13A	0.2885	0.5654	0.5551	0.032*	
C14	0.0109 (6)	0.6469 (3)	0.2776 (4)	0.0296 (12)	
H14A	−0.0401	0.6855	0.2360	0.044*	
H14B	0.1319	0.6527	0.2828	0.044*	
H14C	−0.0301	0.6480	0.3471	0.044*	
C15	0.3208 (6)	0.7181 (2)	0.6698 (4)	0.0217 (11)	
C16	0.2956 (6)	0.7066 (2)	0.7776 (4)	0.0229 (11)	
H16A	0.1862	0.7062	0.8004	0.027*	
C17	0.4327 (6)	0.6958 (2)	0.8495 (4)	0.0248 (11)	
H17A	0.4160	0.6872	0.9203	0.030*	
C18	0.5940 (6)	0.6977 (2)	0.8154 (4)	0.0248 (11)	
C19	0.6226 (6)	0.7092 (3)	0.7092 (4)	0.0242 (11)	
H19A	0.7322	0.7099	0.6867	0.029*	
C20	0.4844 (6)	0.7192 (2)	0.6385 (4)	0.0231 (11)	
H20A	0.5020	0.7270	0.5676	0.028*	

### Atomic displacement parameters (Å^2^)

**Table:**

	*U*^11^	*U*^22^	*U*^33^	*U*^12^	*U*^13^	*U*^23^
S1	0.0271 (6)	0.0151 (7)	0.0242 (7)	0.0003 (5)	−0.0022 (5)	−0.0002 (5)
Cl1	0.0445 (8)	0.0359 (9)	0.0306 (8)	−0.0021 (6)	−0.0061 (6)	0.0026 (6)
Cl2	0.0339 (7)	0.0273 (7)	0.0317 (8)	0.0026 (5)	−0.0097 (6)	−0.0012 (6)
N1	0.029 (2)	0.021 (2)	0.023 (2)	−0.0022 (18)	0.0003 (19)	−0.0006 (18)
O1	0.0359 (19)	0.0165 (19)	0.028 (2)	0.0014 (15)	−0.0052 (16)	0.0039 (15)
O2	0.0245 (17)	0.023 (2)	0.033 (2)	−0.0006 (14)	−0.0059 (15)	0.0014 (16)
O3	0.041 (2)	0.023 (2)	0.025 (2)	0.0034 (16)	−0.0047 (16)	−0.0076 (15)
C1	0.033 (3)	0.033 (3)	0.022 (3)	0.000 (2)	0.003 (2)	0.003 (2)
C2	0.039 (3)	0.033 (3)	0.020 (3)	−0.006 (2)	0.004 (2)	−0.004 (2)
C3	0.039 (3)	0.029 (3)	0.025 (3)	−0.012 (2)	0.008 (2)	−0.003 (2)
C4	0.038 (3)	0.015 (3)	0.041 (3)	−0.003 (2)	0.006 (3)	0.000 (2)
C5	0.024 (3)	0.029 (3)	0.033 (3)	0.000 (2)	0.003 (2)	0.003 (2)
C6	0.037 (3)	0.017 (3)	0.034 (3)	−0.004 (2)	−0.001 (3)	−0.002 (2)
C7	0.035 (3)	0.021 (3)	0.036 (3)	0.000 (2)	0.002 (3)	−0.004 (2)
C8	0.025 (3)	0.024 (3)	0.028 (3)	−0.001 (2)	−0.003 (2)	0.001 (2)
C9	0.040 (3)	0.018 (3)	0.030 (3)	−0.001 (2)	0.002 (2)	−0.002 (2)
C10	0.038 (3)	0.020 (3)	0.036 (3)	0.002 (2)	−0.002 (3)	0.006 (2)
C11	0.031 (3)	0.023 (3)	0.020 (3)	0.001 (2)	0.000 (2)	0.001 (2)
C12	0.030 (3)	0.020 (3)	0.032 (3)	−0.005 (2)	0.003 (2)	−0.004 (2)
C13	0.031 (3)	0.016 (3)	0.033 (3)	0.000 (2)	−0.002 (2)	0.002 (2)
C14	0.043 (3)	0.017 (3)	0.029 (3)	−0.005 (2)	0.001 (2)	−0.001 (2)
C15	0.028 (3)	0.015 (3)	0.021 (3)	−0.001 (2)	0.000 (2)	−0.005 (2)
C16	0.030 (3)	0.010 (2)	0.029 (3)	0.0008 (19)	0.004 (2)	−0.002 (2)
C17	0.037 (3)	0.014 (3)	0.022 (3)	0.002 (2)	−0.007 (2)	−0.001 (2)
C18	0.030 (3)	0.011 (3)	0.032 (3)	0.001 (2)	−0.005 (2)	−0.002 (2)
C19	0.022 (2)	0.023 (3)	0.027 (3)	0.000 (2)	−0.001 (2)	−0.002 (2)
C20	0.029 (3)	0.019 (3)	0.021 (3)	−0.004 (2)	0.000 (2)	−0.002 (2)

### Geometric parameters (Å, °)

**Table:**

S1—O2	1.450 (3)	C8—C9	1.402 (7)
S1—O3	1.451 (3)	C9—C10	1.383 (7)
S1—O1	1.462 (3)	C9—H9A	0.9300
S1—C15	1.781 (5)	C10—C11	1.370 (7)
Cl1—C11	1.741 (5)	C10—H10A	0.9300
Cl2—C18	1.745 (5)	C11—C12	1.389 (7)
N1—C1	1.350 (6)	C12—C13	1.382 (7)
N1—C5	1.375 (6)	C12—H12A	0.9300
N1—C14	1.482 (6)	C13—H13A	0.9300
C1—C2	1.369 (7)	C14—H14A	0.9600
C1—H1A	0.9300	C14—H14B	0.9600
C2—C3	1.370 (7)	C14—H14C	0.9600
C2—H2A	0.9300	C15—C20	1.379 (6)
C3—C4	1.372 (7)	C15—C16	1.411 (6)
C3—H3A	0.9300	C16—C17	1.382 (7)
C4—C5	1.406 (7)	C16—H16A	0.9300
C4—H4A	0.9300	C17—C18	1.374 (7)
C5—C6	1.432 (7)	C17—H17A	0.9300
C6—C7	1.339 (7)	C18—C19	1.398 (7)
C6—H6A	0.9300	C19—C20	1.377 (6)
C7—C8	1.475 (7)	C19—H19A	0.9300
C7—H7A	0.9300	C20—H20A	0.9300
C8—C13	1.397 (7)		
			
O2—S1—O3	113.6 (2)	C11—C10—C9	119.4 (5)
O2—S1—O1	112.9 (2)	C11—C10—H10A	120.3
O3—S1—O1	113.3 (2)	C9—C10—H10A	120.3
O2—S1—C15	105.5 (2)	C10—C11—C12	121.5 (5)
O3—S1—C15	104.1 (2)	C10—C11—Cl1	118.6 (4)
O1—S1—C15	106.4 (2)	C12—C11—Cl1	119.8 (4)
C1—N1—C5	122.0 (4)	C13—C12—C11	118.7 (5)
C1—N1—C14	117.4 (4)	C13—C12—H12A	120.7
C5—N1—C14	120.5 (4)	C11—C12—H12A	120.7
N1—C1—C2	121.1 (5)	C12—C13—C8	121.4 (5)
N1—C1—H1A	119.4	C12—C13—H13A	119.3
C2—C1—H1A	119.4	C8—C13—H13A	119.3
C1—C2—C3	119.0 (5)	N1—C14—H14A	109.5
C1—C2—H2A	120.5	N1—C14—H14B	109.5
C3—C2—H2A	120.5	H14A—C14—H14B	109.5
C2—C3—C4	120.2 (5)	N1—C14—H14C	109.5
C2—C3—H3A	119.9	H14A—C14—H14C	109.5
C4—C3—H3A	119.9	H14B—C14—H14C	109.5
C3—C4—C5	121.2 (5)	C20—C15—C16	118.6 (4)
C3—C4—H4A	119.4	C20—C15—S1	119.6 (4)
C5—C4—H4A	119.4	C16—C15—S1	121.8 (4)
N1—C5—C4	116.6 (5)	C17—C16—C15	120.3 (4)
N1—C5—C6	118.2 (4)	C17—C16—H16A	119.8
C4—C5—C6	125.2 (5)	C15—C16—H16A	119.8
C7—C6—C5	123.1 (5)	C18—C17—C16	119.4 (5)
C7—C6—H6A	118.4	C18—C17—H17A	120.3
C5—C6—H6A	118.4	C16—C17—H17A	120.3
C6—C7—C8	125.5 (5)	C17—C18—C19	121.5 (4)
C6—C7—H7A	117.2	C17—C18—Cl2	120.1 (4)
C8—C7—H7A	117.2	C19—C18—Cl2	118.4 (4)
C13—C8—C9	118.1 (5)	C20—C19—C18	118.3 (4)
C13—C8—C7	123.3 (5)	C20—C19—H19A	120.8
C9—C8—C7	118.6 (5)	C18—C19—H19A	120.8
C10—C9—C8	120.9 (5)	C19—C20—C15	121.9 (4)
C10—C9—H9A	119.6	C19—C20—H20A	119.1
C8—C9—H9A	119.6	C15—C20—H20A	119.1
			
C5—N1—C1—C2	−0.6 (7)	C10—C11—C12—C13	0.6 (7)
C14—N1—C1—C2	178.0 (4)	Cl1—C11—C12—C13	−179.1 (4)
N1—C1—C2—C3	−0.3 (7)	C11—C12—C13—C8	0.3 (7)
C1—C2—C3—C4	1.1 (7)	C9—C8—C13—C12	−0.3 (7)
C2—C3—C4—C5	−1.1 (7)	C7—C8—C13—C12	−179.8 (4)
C1—N1—C5—C4	0.6 (6)	O2—S1—C15—C20	165.8 (4)
C14—N1—C5—C4	−177.9 (4)	O3—S1—C15—C20	45.9 (4)
C1—N1—C5—C6	178.3 (4)	O1—S1—C15—C20	−74.1 (4)
C14—N1—C5—C6	−0.3 (6)	O2—S1—C15—C16	−14.1 (4)
C3—C4—C5—N1	0.2 (7)	O3—S1—C15—C16	−134.0 (4)
C3—C4—C5—C6	−177.3 (5)	O1—S1—C15—C16	106.0 (4)
N1—C5—C6—C7	174.7 (5)	C20—C15—C16—C17	−0.8 (7)
C4—C5—C6—C7	−7.9 (8)	S1—C15—C16—C17	179.1 (4)
C5—C6—C7—C8	178.2 (4)	C15—C16—C17—C18	1.2 (7)
C6—C7—C8—C13	7.1 (8)	C16—C17—C18—C19	−1.1 (7)
C6—C7—C8—C9	−172.5 (5)	C16—C17—C18—Cl2	177.2 (4)
C13—C8—C9—C10	−0.7 (7)	C17—C18—C19—C20	0.6 (7)
C7—C8—C9—C10	178.9 (4)	Cl2—C18—C19—C20	−177.7 (4)
C8—C9—C10—C11	1.6 (7)	C18—C19—C20—C15	−0.2 (7)
C9—C10—C11—C12	−1.6 (7)	C16—C15—C20—C19	0.3 (7)
C9—C10—C11—Cl1	178.2 (4)	S1—C15—C20—C19	−179.6 (4)

### Hydrogen-bond geometry (Å, °)

**Table:**

*D*—H···*A*	*D*—H	H···*A*	*D*···*A*	*D*—H···*A*
C1—H1A···Cl2^i^	0.93	2.81	3.572 (5)	140
C3—H3A···O1^ii^	0.93	2.51	3.411 (6)	163
C6—H6A···O3	0.93	2.55	3.467 (6)	168
C7—H7A···O2^iii^	0.93	2.48	3.395 (6)	166
C13—H13A···O3	0.93	2.48	3.413 (6)	178
C14—H14A···O2^iv^	0.96	2.44	3.292 (6)	148
C14—H14C···O3	0.96	2.48	2.995 (6)	114
C16—H16A···O2	0.93	2.58	2.936 (6)	103
C19—H19A···O2^v^	0.93	2.29	3.216 (6)	178
C10—H10A···Cg3^vi^	0.93	2.71	3.632 (6)	172
C12—H12A···Cg3	0.93	2.75	3.614 (6)	154

Symmetry codes: (i) *x*−1, *y*, *z*−1; (ii) −*x*, *y*−1/2, −*z*+1/2;  (iii) −*x*, −*y*+1, −*z*+1; (iv) *x*, −*y*+3/2, *z*−1/2; (v) *x*+1, *y*, *z*; (vi) −*x*+1, *y*−1/2, −*z*+3/2.
